# Supplementary material for: Combinative effects of Azospirillum brasilense inoculation and chemical priming on germination behavior and seedling growth in aged grass seeds
Source: PLoS One. 2019 May 7;14(5):e0210453. doi: 10.1371/journal.pone.0210453 (PMC6504077; doi:10.1371/journal.pone.0210453)
Supplement: S1 File — Table A. The MDA content and the activity of SOD and POD in tall fescue 2015, 2014, orchardgrass 2013 and Russian wild rye 2009. Table B. Canonical response surface analysis for germination percentage, germination index, seed vigor index, root, shoot and seedling length, ratio of S/R, MDA content, the activity of SOD, POD, CAT and APX of the seedlings based on coded data. Table C. Pearson correlation coefficients for activity of SOD, APX, POD, CAT, and MDA content. (PDF) [file pone.0210453.s001.pdf]

Supplementary Material S1

Combinative effects of *Azospirillum brasilense* inoculation and chemical priming on germination behavior and seedling growth in aged grass seeds

Xu Liu[*], Zhao Chen[1], Yani Gao[1], Qian Liu[1], Wennan Zhou[1], Tian Zhao[1], Wenbo Jiang[1], Xuewen Cui[1], Jian Cui[2] and Quanzhen Wang[1]

***Correspondence:** Quanzhen Wang: wangquanzhen191@163.com

Table A. The MDA content and the activity of SOD and POD in tall fescue 2015, 2014, orchardgrass 2013 and Russian wild rye 2009.

**Species**	**MDA** (μmol g^{-1} FW)	**SOD** (U/g.FW/h)	**POD (0.000)** (U/g.FW/min)
Tall fescue 2015	13.80±5.22[b]	17.06±8.83[b]	1.62±0.49[c]
Tall fescue 2014	9.94±0.22[b]	12.20±8.21[b]	1.33±0.64[c]
Orchardgrass 2013	11.01±2.43[b]	25.63±14.24[a]	3.17±1.18 [a]
Russian wildrye 2009	37.76±9.73[a]	5.43±14.32[c]	2.37±0.78 [b]

†Data are the mean ± SD.

‡Means followed by different letters are significantly different at $P < 0.05$.

Table B. Canonical response surface analysis for germination percentage, germination index, seed vigor index, root, shoot and seedling length, ratio of S/R, MDA content, the activity of SOD, POD, CAT and APX of the seedlings based on coded data.

Variable	**Critical value of bacteria (X_1)**	**Critical value of time (X_3)**	*F* Value	*Pr>F*	Estimated value	Stationary point
**Tall fescue 2015**						
Germination percentage	**50.6**	**19.1**	3.04	0.036		minimum
Germination index	**50.1**	**20.5**	2.87	0.036		saddle point
Seed vigor index	**63.2**	**6.9**	3.08	0.028		maximum
Root length	**49.7**	**16.4**	2.31	0.076		maximum
Ratio of shoot to root	**54.7**	**17.5**	3.48	0.017		minimum
SOD activity	**38.2**	**7.4**	2.77	0.041		saddle point
MDA content	**55.8**	**18.0**	3.31	0.020		saddle point
CAT content	**49.5**	**24.8**	9.31	<.0001		saddle point
APX activity	**52.1**	**15.6**	4.21	0.007		minimum
**Tall fescue 2014**						
Seed vigor index	**65.4**	**11.6**	2.68	0.047		maximum
Root length	**59.0**	**16.0**	5.76	0.001		saddle point
Seedling length	**51.0**	**26.3**	4.42	0.005		maximum
Ratio of shoot to root	**41.0**	**32.0**	7.77	<.0001		minimum
MDA content	**52.8**	**19.1**	6.73	<.0001		saddle point
**Orchardgrass 2013**						
Germination index	**51.1**	**14.4**	3.19	0.024		saddle point
Seed vigor index	**48.6**	**13.8**	2.965	0.032		saddle point
Root length	**45.4**	**5.9**	3.51	0.016		maximum
Seedling length	**55.7**	**6.2**	2.95	0.032		maximum
Ratio of shoot to root	**52.9**	**5.7**	5.73	0.001		minimum
SOD activity	**53.1**	**13.9**	10.72	<.0001		saddle point
POD activity	**23.5**	**20.0**	6.84	<.0001		saddle point
CAT content	**41.3**	**12.2**	7.89	<.0001		saddle point
APX activity	**19.6**	**44.9**	7.00	0.0002		saddle point
**Russian wildrye 2009**						
Germination percentage	**57.8**	**13.0**	9.30	<.0001		saddle point
Germination index	**52.5**	**14.4**	3.19	0.024		saddle point
Seed vigor index	**54.2**	**9.2**	4.46	0.005		maximum
Root length	**52.9**	**20.0**	3.15	0.025		maximum
Shoot length	**55.5**	**17.3**	5.58	0.0015		maximum
Seedling length	**53.8**	**18.0**	5.516	0.0016		maximum
POD activity	**61.1**	**3.9**	2.82	0.038		maximum
MDA content	**52.8**	**19.2**	7.44	0.0008		minimum
CAT content	**47.6**	**19.4**	4.53	0.005		saddle point

Table C. Pearson correlation coefficients for activity of SOD, APX, POD, CAT, and MDA content.

	Germination percentage	Root length	SOD	APX	POD	CAT	MDA
Germination percentage	1	0.323**	0.419**	-0.378**	-0.262**	-0.140	-0.519**
Root length		1	0.212**	-0.445**	-0.447**	-0.194**	-0.450**
SOD			1	-0.262**	-0.041	0.045	-0.249**
APX				1	0.452**	0.165	0.373**
POD					1	0.119	0.383**
CAT						1	0.216*
MDA							1

*Significant at the 0.05 probability level.

**Significant at the 0.01 probability level.
